# Assessment of glove integrity across various dental specialties in a dental school setting

**DOI:** 10.3389/froh.2024.1496918

**Published:** 2024-12-18

**Authors:** Mohammed Amjed Alsaegh, Mohammed Farooq AlSiraj, Ahmed Naji Alsadoon, Omar Soufi, Okba Mahmoud, Sudhir Rama Varma

**Affiliations:** ^1^ Department of Oral and Craniofacial Health Sciences, College of Dental Medicine, University of Sharjah, Sharjah, United Arab Emirates; ^2^ Department of Clinical Sciences, College of Dentistry, Ajman University, Ajman, United Arab Emirates; ^3^ Center for Medical and Bio-allied Health Sciences Research, Ajman University, Ajman, United Arab Emirates

**Keywords:** gloves, latex, integrity, perforation, dental school

## Abstract

**Objectives:**

This study aimed to evaluate the integrity of non-sterile, powder-free latex gloves used by dental students in various dental specialties.

**Materials and methods:**

This cross-sectional study involved dental students from Ajman University who provided gloves during various dental specialty procedures. A total of 177 pairs of latex examination powder-free gloves were included and categorized as follows: 43 pairs (24.3%) were used in operative dentistry, 30 pairs (16.9%) in oral surgery, 28 pairs (15.8%) in endodontics, 24 pairs (13.6%) in periodontics, 21 pairs (11.9%) in pedodontics, 13 pairs (7.3%) in prosthodontics, and 18 pairs (10.2%) as control gloves. After use, glove integrity was assessed with a modified water leak test.

**Results:**

Perforations were identified in 72 cases (40.7%) of gloves, distributed as follows: 22 cases (51.2%) in operative dentistry, 12 cases (40.0%) in oral surgery, 11 cases (39.3%) in endodontics, 11 cases (45.8%) in periodontics, 10 cases (47.6%) in pedodontics, 5 cases (38.5%) in prosthodontics, and 1 case (5.5%) in the control group. There were no statistically significant differences in the loss of glove integrity among different dental specialty procedures (χ^2^ = 11.899, *p* = 0.064) or among different glove usage durations (χ^2^ = 1.732, *p* = 0.785). However, the location of perforations in the experimental groups was statistically significant (χ^2^ = 34.427, *p* < 0.001). The most common locations were the right thumb (*n* = 18; 13.7%) and the right index finger (*n* = 17; 13%), with no perforations in the left ring finger and only one perforation in the left little finger (*n* = 1, 0.08%). There was a statistically significant correlation between the anticipated and actual presence of defects (χ^2^ = 32.875, *p* < 0.001).

**Conclusions:**

The study found a high rate of glove perforations during dental procedures by undergraduate students, especially in the right thumb and index finger. To reduce cross-infection risks, strict protocols like double gloving, frequent glove changes, and covering wounds with plaster are recommended.

## Introduction

In today's healthcare environment, there is heightened concern among medical professionals about the transmission of deadly viruses such as HIV, hepatitis, and SARS-CoV-2 from patients. This has led to a renewed emphasis on personal protective equipment (PPE), including medical gloves. The risk of infection after percutaneous exposure to HIV, hepatitis B, and hepatitis C viruses varies widely (1).

Clinical staff, including physicians and dentists, are required to wear medical gloves to protect both themselves and their patients from contamination. There are two main types of medical gloves: examination, or procedure gloves for routine examinations and minor procedures, and surgical gloves for use during surgery. Examination gloves are crucial component of PPE for healthcare workers (2).

Typically made from latex, procedure gloves are recommended for semi-critical procedures where the vascular system is not invaded. They are single-use items, and a new pair should be used for each patient and then disposed of. Surgical gloves, used in conjunction with surgical hand antisepsis, are essential but do not guarantee complete protection due to the frequent occurrence of micro perforations or tears, which often go unnoticed by users, exposing both the patient and healthcare worker to potential infections (3).

Medical gloves can be made of latex, nitrile, or vinyl (4). Latex and nitrile gloves are comparable in terms of barrier protection (5). However, nitrile gloves are less elastic (5, 6), which can reduce dexterity for fine motor tasks (6). It has been found that nitrile and latex gloves offer better barrier protection than vinyl gloves, which show decreased durability and potentially compromise barrier protection (4).

Despite the type of glove used, perforations are common in clinical settings (7). Dental practitioners, in particular, are at risk of infection due to the nature of their work, which involves frequent contact with sharp instruments, often at high speeds, and working in the oral cavity with contaminated fluids like saliva and blood. Many dental interns and postgraduate students lack adequate knowledge of proper glove use, highlighting the need for better education and training in infection control practices (8).

There is limited literature on the frequency and location of glove tears during dental procedures, particularly those performed by dental students. Furthermore, the limited studies available reported varying findings on the prevalence of glove perforations during dental procedures (9–11). The current study aimed to assess the integrity of non-sterile, powder-free latex gloves used by dental students during various dental specialty procedures.

## Materials and methods

**Figure 1:**
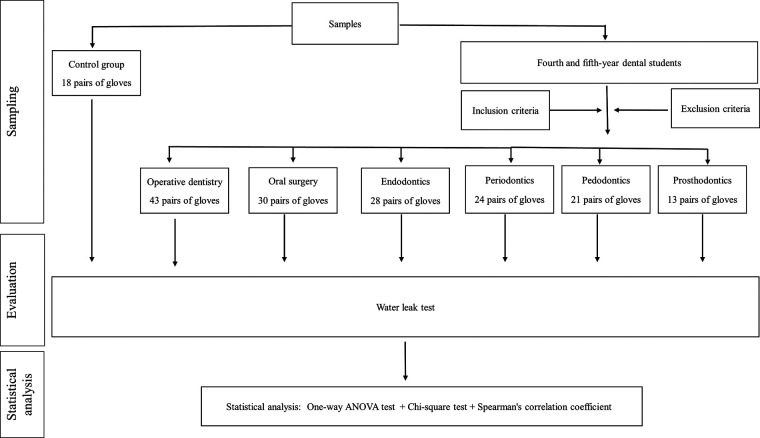
A diagrammatic representation of the chronology of the methodology.

This cross-sectional study evaluated 177 pairs of ambidextrous, powder-free, disposable latex examination gloves (Masterguard, Malaysia), totaling 354 single gloves. The participants were randomly selected fourth and fifth-year dental students from Ajman University (Figure 1). Each participant provided consent and completed a questionnaire form. Following a strict infection control protocol, each student was given a pair of gloves. The participants recorded data immediately after discarding the gloves, including dental specialty clinics, duration of the procedure, dominant hand, and any anticipated glove perforations. A 10% random sample from each opened pack of gloves was selected, resulting in a control group of 36 gloves. This study adhered to the Declaration of Helsinki principles and was approved by the Research Ethical Committee of Ajman University (Reference No.: UGD-H-18-11-22-17).

The sample size was calculated using G*Power software (ver. 3.1.9.7; University of Düsseldorf, Germany), based on da Thomson et al. (12). information, with an effect size of 0.2, 80% power, and a 5% error rate, resulting in a requirement for 321 samples. We selected as a convenience sample and get 354 samples for this study.

Inclusion criteria included routine dental procedures performed by fourth and fifth-year dental students. The used gloves were in suitable condition as determined by visual inspection, and procedures lasting between 15 and 180 min. Exclusion criteria included gloves with folds or defects, gloves tearing during wear or removal, students with latex allergies, or gloves with abnormal viscosity.

**Figure 2:**
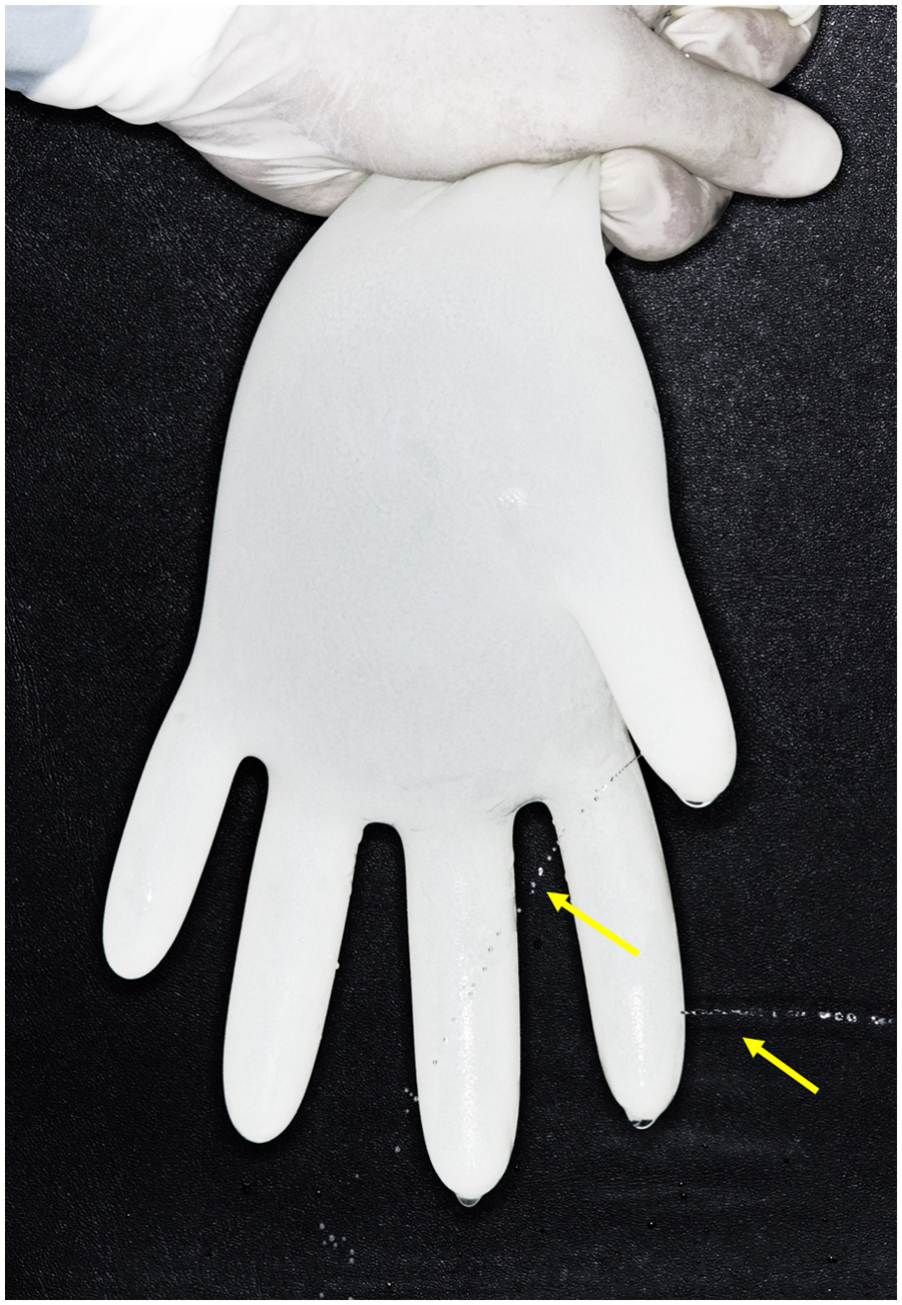
Modified water leak test used in the current study to identify glove perforations. Arrows indicate water leakage resulting from perforations in the index and thumb finger areas.

Participants used the gloves as usual, then carefully doffed them to avoid tearing and discarded them in coded bags. Gloves in various sizes are readily available, allowing each student participant to select a pair that fits comfortably, ensuring they are neither too loose nor too tight. To ensure stringent infection control, fingernails were kept short with smooth, filed edges to facilitate thorough cleaning and reduce glove tears risk. Hand and nail jewelry were not allowed. Prior to donning gloves for any procedure, hand washing, and surgical antisepsis were performed using antimicrobial soap and water to maintain hygiene standards. Following each treatment, gloved hands were cleaned with Hibiscrub solution (Mölnlycke Health Care, Gothenburg, Sweden). The gloves were then removed with care and placed in labeled plastic bags, each marked with a unique identifier corresponding to the attached questionnaire. Glove integrity was assessed using a modification of the previously documented standard water leak test method (12). Gloves were filled with 500 ml of water, held against a dark background, and observed for punctures under gentle pressure for 60 s (Figure 2). The number and location of punctures were recorded on a chart, and control samples were tested similarly.

Data was collected, tabulated, and analyzed using SPSS ver. 28 (IBM Corporation, Armonk, NY, USA). Quantitative variables were presented as mean, standard deviation, coefficient of variation, range, and standard error. A one-way ANOVA test compared the means between groups, and a Chi-square test analyzed categorical variables. Spearman's correlation coefficient determined correlations between independent variables, with significance set at *P* = 0.05.

## Results

A total of 177 pairs of latex examination gloves were analyzed. The distribution was as follows: 43 pairs (24.3%) were used in operative dentistry, 30 pairs (16.9%) in oral surgery, 28 pairs (15.8%) in endodontics, 24 pairs (13.6%) in periodontics, 21 pairs (11.9%) in pedodontics, 13 pairs (7.3%) in prosthodontics, and 18 pairs (10.2%) served as a control group. The experimental groups comprised gloves used by fourth and fifth-year18 dental students, including 94 males (59.1%) and 65 females (40.9%) (Table 1).

**Table 1:** Demographic data of the study samples.

Variables	Experimental group	Control group
Samples (*n* = 177)	152 (89.8%)	18 (10.2%)
Gender, *n* (%)		
Male	94 (59.1%) males	
Female	65 (40.9%) females	
Dental specialties	Operative procedures = 43 (24.3%)	18 (10.2%)
	Oral surgery = 30 (16.9%)	
	Endodontics = 28 (15.8%)	
	Periodontics = 24 (13.6%)	
	Pedodontics = 21 (11.9%)	
	Prosthodontics = 13 (7.3%)	

**Figure 3:**
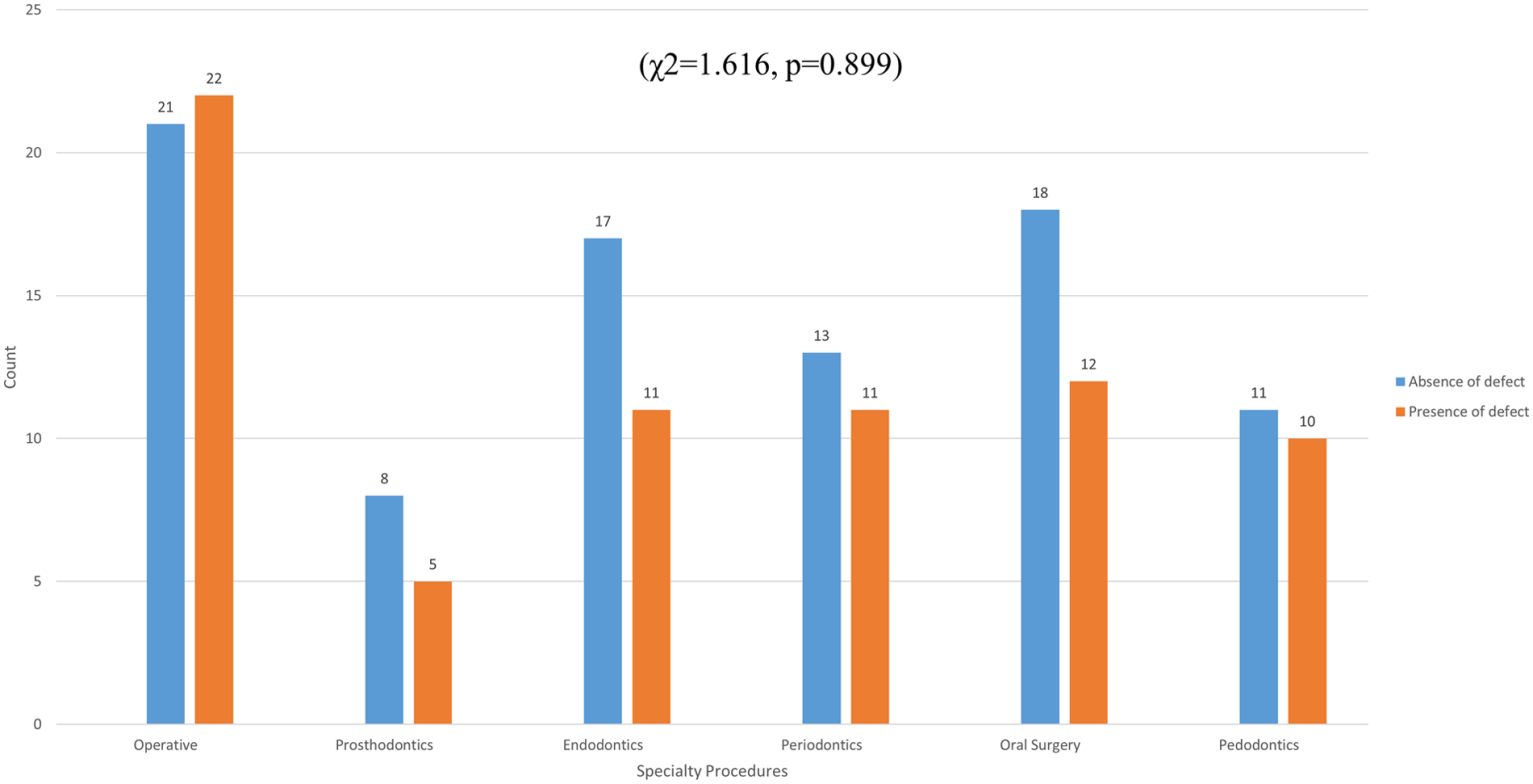
The incidence of glove integrity loss across various dental specialty procedures.

Glove perforations were found in 72 cases (40.7%) of gloves, distributed as follows: 22 cases (51.2%) in operative dentistry, 12 cases (40.0%) in oral surgery, 11 cases (39.3%) in endodontics, 11 cases (45.8%) in periodontics, 10 cases (47.6%) in pedodontics, 5 cases (38.5%) in prosthodontics, and 1 case (5.5%) in the control group (Figure 3).

**Figure 4:**
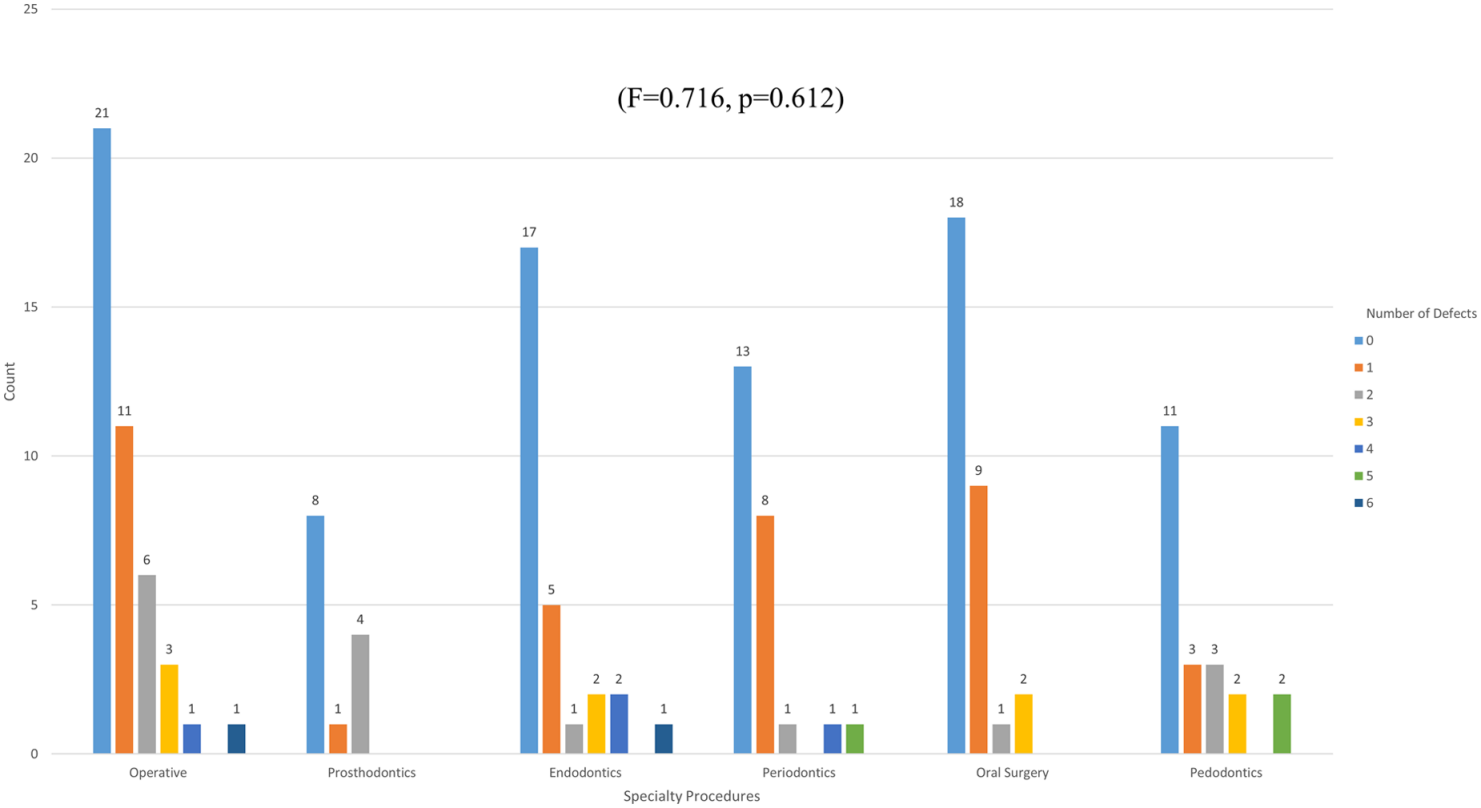
The incidence of numbers of glove defects across various dental specialty procedures.

There was a statistically significant difference between the experimental and control groups (χ^2^ = 10.243, *p* = 0.001). However, the presence of perforation among different dental specialty procedures was not statistically significant (χ^2^ = 1.616, *p* = 0.899). One-way ANOVA statistical analysis showed non-significant differences in the number of defects in the glove samples among different dental specialty procedures (F = 0.716, *p* = 0.612) (Figure 4).

**Figure 5:**
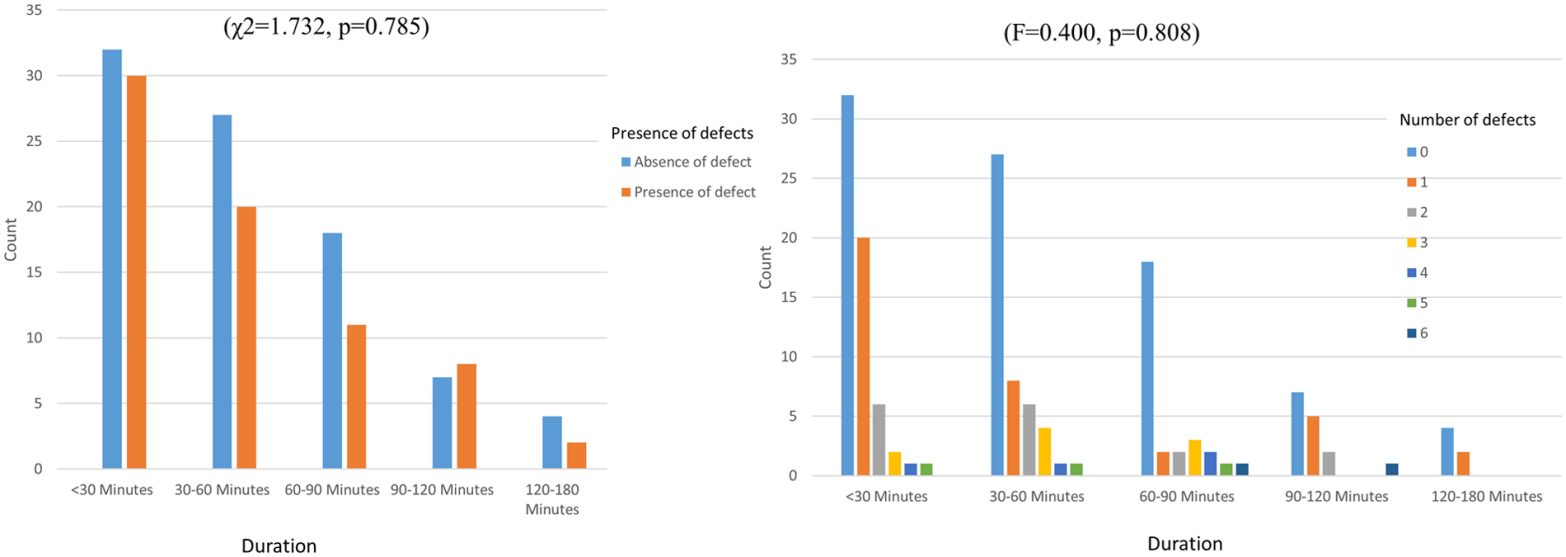
Incidence and number of glove integrity defects across different durations of use.

The study revealed no significant variation in glove integrity loss across different durations of use (χ^2^ = 1.732, *p* = 0.785) (Figure 4). Additionally, there was no significant difference in the number of defects relative to the duration of dental treatment (F = 0.400, *p* = 0.808) (Figure 5).

**Figure 6:**
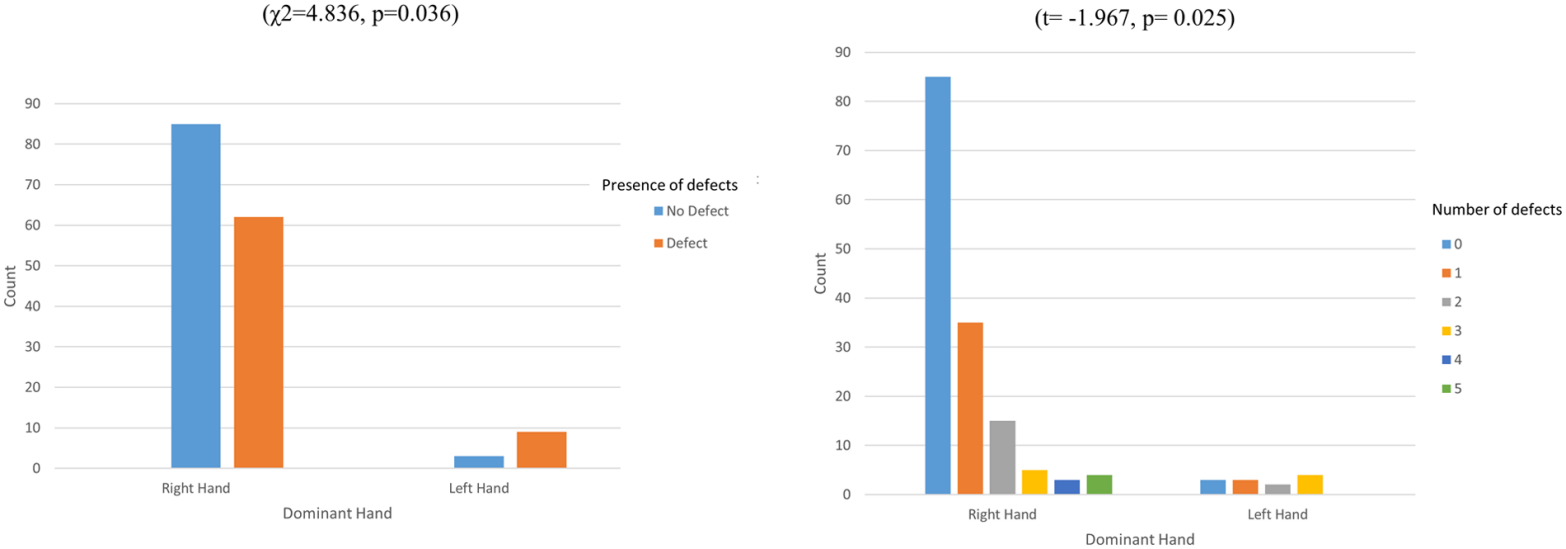
Differences in the incidence and number of glove integrity defects based on participants’ dominant hand.

Participants with left-hand dominance exhibited a significantly higher perforation rate compared to those with right-hand dominance (χ^2^ = 4.836, *p* = 0.036). Specifically, 62 right-handed participants (42.2%) experienced glove defects, while 9 left-handed participants (75%) encountered glove defects. Additionally, a statistically significant difference (*t* = −1.967, *p* = 0.025) was observed in the number of perforations between participants with right and left dominant hands (Figure 6).

The location of perforations in the experimental groups was statistically significant (χ^2^ = 34.427, *p* < 0.001). The most common perforation locations were the right thumb (*n* = 18; 13.7%) and the right index finger (*n* = 17; 13%), with no perforations in the left ring finger and only one in the left little finger (*n* = 1; 0.08%) (Table 2). There was no correlation between the location of glove perforations and the various dental specialties (rho = 0.025, *p* = 0.781), nor between the dominant hand and the perforation location (χ^2^ = 11.953, *p* = 0.532). Right-hand gloves exhibited higher perforation rates regardless of dominant hand (Table 2).

**Table 2:** The location of perforations in the dominant and non-dominant hands of the operators.

		Right thumb	Right index finger	Right middle finger	Right ring finger	Right little finger	Right palm	Right back side	Left thumb	Left index finger	Left middle finger	Left little finger	Left palm	Left back side
Dominant Hand	Right Hand	14	15	8	7	2	7	13	13	11	4	0	9	8
	Left Hand	4	2	0	1	1	3	2	2	2	0	1	1	1
Total		18	17	8	8	3	10	15	15	13	4	1	10	9

The anticipation of glove integrity matched the actual integrity in 100 cases (62.9%). However, there were 42 cases (26.4%) where perforations were not anticipated and 17 cases (10.7%) where perforations were suspected but not present. Despite the high percentage of mismatches, the correlation between anticipated and actual defects was statistically significant (χ^2^ = 32.875, *p* < 0.001). Lastly, there was no significant difference in loss of integrity of the used gloves between male and female participants (χ^2^ = 0.432, *p* = 0.52) (Table 3).

**Table 3:** Glove integrity loss during various dental specialty procedures among male and female dental students.

		Presence of defect		Total
		Not present	Present	
Gender	Male	*n* = 50	*n* = 44	*n* = 94
	Female	*n* = 38	*n* = 27	*n* = 65
Total		*n* = 88	*n* = 71	*n* = 159

## Discussion

In the current study, a relatively high rate of glove perforation was observed among dental students during various dental specialty procedures. This finding aligns with previous studies reporting high perforation rates, such as 41.4% in gloves used in emergency procedures, 30.0% in elective surgeries, and 41.4% in major medical surgeries (13) as well as 41.4% (14) and 52% (15) in orthopedic surgeries. Contrary, lower perforation rates have been reported in other studies, such as 1.8% during hand surgeries (16), 5.9% in typical orthopedic trauma procedures (12), and 27.1% in clinical dental practice (17). Burke et al. reported a 16% perforation rate after dental extractions (9). In prosthodontic procedures, Nikawa et al. detected latex glove perforation in 27.9% (10). Avery et al. found a perforation rate of 4.3%–8.6% per surgeon and operative site after wisdom teeth extractions (11). Differences in the rate of glove integrity loss across studies may be partially due to the type and quality of gloves used, the type of procedure, or the clinician's experience.

The high rate of integrity loss underscores the need for innovation in glove materials to improve durability and reduce the risk of failure, enhancing overall safety for both dental professionals and patients. The integrity of gloves used by medical practitioners is crucial, especially during pandemics such as COVID-19.

The high glove perforation rate in the current study may be attributed to the type of gloves used. Our samples were latex examination gloves, commonly used for straightforward dental procedures where infection control is not as strict. Indeed, sales of latex-containing gloves are decreasing, benefiting dental practitioners and latex-allergic patients (18). Examination gloves are generally less standardized than surgical gloves, with many manufacturers only testing samples of gloves from each batch for leaks (19). Pinholes in gloves can be minimized through manufacturing processes involving high curing temperatures, high oven temperatures before coagulation, and other methods (20). Pitten et al. found a 5.4% perforation rate in samples of the control group (17), which aligns with our study, where 5.5% of control samples showed glove integrity loss. However, another study reported a penetration rate as high as 16.1% in unused gloves (21). Numerous national and international standards for quality control in dental procedure gloves are essential to ensure patient safety by preserving the gloves' integrity and effectiveness as a barrier against infections. These standards include various tests and guidelines to evaluate the physical and microbiological properties of gloves, ensuring they provide reliable protection during dental procedures. Their primary goal is to prevent cross-contamination and safeguard both patients and healthcare professionals from infectious agents. Key features outlined in international standards for medical safety gloves include freedom from holes, appropriate dimensions, physical properties such as force at break and tensile strength, protein leach levels, powder content, and shelf life (22). Organizations like the European Committee for Standardization (CEN) and the International Organization for Standardization (ISO) play a significant role in developing these glove standards.

Several factors contribute to the diminished integrity of latex gloves, such as prolonged use, humidity, intense manipulation of instruments, and exposure to chemical products (23). Despite these limitations, latex gloves have shown better resistance to perforation compared to vinyl and nitrile gloves (4, 24). An experiment demonstrated that latex had significantly better bacterial barrier efficacy compared to nitrile gloves in the presence of micro perforations (25). Furthermore, the high perforation rate in gloves used in dentistry could be attributed to the chemicals used during dental procedures (26). Another factor contributing to the high perforation rate could be the mechanical factor of dental procedures, characterized by intensive hand use, generating friction and stress in the fingers.

In the present study, the participants were fourth- and fifth-year undergraduate dental students, considered novice practitioners. Their lack of experience may have contributed to the higher glove perforation rates observed. A previous study assessed and compared glove perforation rates between primary surgeons and assistant surgeons, reporting a significantly higher incidence of perforations in gloves worn by primary surgeons (27). However, another study by Feng et al. found no significant difference in glove perforation rates between primary surgeons and assistants (28).

Previous studies recommended waiting until hands are fully dry before donning a new pair of gloves to reduce perforation rates (17). In addition, wearing properly fitted gloves is crucial for maintaining integrity. Gloves that don't fit properly can lead to higher perforation rates. Ensuring a variety of glove sizes are available can decrease perforation likelihood, enhancing safety for healthcare workers and patients. Tight gloves restrict movement and may tear due to stretching, while loose gloves can get caught in equipment or tissues during procedures (1). A previous study found that the perforation rates for properly fitting, tight, and loose medical gloves were 20%, 37.78%, and 34.81%, respectively, indicating that wearing the wrong size gloves may increase the likelihood of perforation (1).

Double gloving has been shown to protect surgeons' hands in 80.4% of cases where glove integrity was compromised, with the inner glove remaining undamaged (14). A previous systematic review demonstrated that double gloving reduces surgical glove perforation rates and decreases the risk of contamination by blood-borne pathogens (29). However, the difference in perforation rates between single- and double-gloved procedures is not always significant (16). Moreover, Johnson et al. found that dexterity decreases with thicker gloves, suggesting that multiple layers may restrict movement (30). Despite evidence supporting double gloving, many surgeons do not practice it regularly. Enhanced education on the benefits of double gloving and early introduction of this practice could increase its adoption (31).

Changing gloves during long procedures can also reduce perforations. It has been suggested that changing gloves as early as 15 min from the start provides a good balance between feasibility and safety (32), while another study recommended changing gloves every 90 min (3).

Despite a higher loss of glove integrity during operative procedures, the study found non-significant differences in glove integrity across various dental specialty procedures. This suggests that glove quality and material, rather than the specific specialty, might be the main factors contributing to the high loss of integrity of the gloves. While dental procedures vary in manipulation and instrument use, standardized techniques and precautions likely result in a uniform rate of glove integrity loss. Accordingly, the high frequency of glove perforations in operative dentistry could be due to the instruments used. A study of orthopedic surgery procedures found that 52% of glove perforations were caused by drills, reamers, K wires, and other instruments (15).

Our study did not reveal any correlation between the operator's gender and the integrity loss of the gloves used. Interestingly, it was observed that the length of clinicians' fingernails significantly compromises the integrity of latex gloves. Therefore, maintaining short fingernails is crucial for reducing the risk of glove damage during surgical procedures (33).

The current study found no difference in loss of glove integrity across various durations of use. Additionally, there was no difference in the number of glove defects related to the length of dental procedures. This finding is consistent with previous studies conducted on dental procedures (17), general surgery (34), abdominal surgery (35), and urology (28). However, it has been found that operation times of more than one hour increased the risk of perforation by 12.77 times during open abdominal surgeries (36) and by about three times in orthopedic trauma procedures (14). In addition, another study found that the majority of perforated gloves were detected when the duration of the procedure exceeded 90 min in maxillofacial, urology, and general and digestive surgeries (37).

Possibly, the lack of a relation between glove perforations and the duration of the dental procedure in the current study can be attributed to the high perforation rate, which masks the effect of time on perforations. Nevertheless, discrepancies in previous studies regarding the association between procedure duration and glove perforation could be due to differences in sample types, surgical techniques and skill levels, and glove types and quality. For example, a previous report in orthopedic surgery found that the correlation between the number of damaged gloves and surgery duration was significant in revision arthroplasties but not in primary endoprosthetics (38).

These conflicting results have led to different recommendations for the timing of surgical glove replacement to preserve glove integrity (39). The tendency has been to consider that longer surgeries increase the risk of perforation; thus, it has been suggested that gloves be replaced during procedures lasting 60–120 min (40). Replacing gloves during surgeries lasting over two hours is important not only to reduce the possibility of contaminating the open surgical wound but also to minimize latex wear, depending on the type of surgery, thereby decreasing the perforation rate (39).

Our study identified statistically significant variation in the location of glove perforations within the experimental groups. The most frequent site of perforation was the right thumb, followed by the right index finger, while no perforations were observed in the left ring finger, and only one instance of perforation was noted in the left little finger across all samples. Additionally, there was no correlation between the location of glove integrity loss and the specific dental specialty procedures performed. Furthermore, no association was found between hand dominance and the location of perforations.

A previous study found that most glove perforations occur on surgeons' index fingers (7, 14, 16), with 75% on the dominant hand (16). Other studies found that the highest incidence of perforations was on the index finger of the non-dominant hand among various surgical teams, including gastroenterological, cardiovascular, and pediatric teams (39), as well as in open surgeries (34, 36) and in urology, maxillofacial, and general and digestive surgeries (37). Interestingly, in simulated dental procedures, perforations were predominantly observed on the index fingers and thumbs (33).

The increased damage in these areas was attributed to their frequent use, greater involvement with sharp objects, and higher contact with patient tissues (41). The type of surgical procedure may influence the localization of glove defects. Significant differences in the position of glove damage were observed across different orthopedic surgeries (38).

In the present study, right-hand gloves exhibited a higher number of perforations regardless of hand dominance. We also found that the left dominant hand's operator had a higher perforation rate than the right dominant hand. Additionally, no correlation was found between hand dominance and the location of perforation in the current study. It was noted that 86% of perforations occurred in the non-dominant hand during visceral surgery (3). In contrast, another previous study reported no difference in perforation rates between gloves worn on either hand during urology surgeries (28). We suggest that the statistical significance of the defect occurrence and hand dominance in our study may be attributed to the small subgroup of left-dominant individuals, which comprised only 12 participants. The results of the current study indicate that special attention should be given to the integrity of gloves at the thumb and index fingers. Further recommendations could involve advising glove manufacturers to evaluate the feasibility of providing enhanced material resistance to perforation in these specific areas.

Despite a high percentage of incorrect anticipations, the findings revealed that the rate of correctly anticipated glove defects was statistically significant. This can be attributed to the operators' heightened sense of awareness regarding potential hazards, making them more attuned to the tactile feedback provided by their gloves, along with high situational awareness. Contrary to our findings, most previous studies have shown that nearly all damages remained unnoticed intraoperatively during various clinical procedures (3, 11, 16, 34, 36). However, it has been reported that 37.5% of glove perforations were recognized by users at the time of occurrence in gastroenterological, cardiovascular, and pediatric surgical teams (39).

This study offers valuable insights into glove integrity loss during dental procedures, updating the available outdated reports, reflecting advances in glove manufacturing, but the study also has several limitations. One limitation is that the study was conducted at a single dental hospital. Additionally, the gloves evaluated were sourced from just one company, which limited the diversity of gloves represented in the market. Furthermore, only latex examination gloves were evaluated, which limits the applicability of the results to other types of disposable gloves. Although reported to be very sensitive, the watertight test used for glove perforations detection in the current study cannot detect perforations smaller than 0.5 mm in diameter (39). Variations in the number of gloves collected during each dental procedure may limit the scope of the statistical analysis. Furthermore, the study only examined gloves used by dental undergraduate students, without comparing them to those used by licensed and specialist dentists.

## Conclusion

The study concluded that glove perforation rates were notably high during dental procedures conducted by undergraduate students. These perforation rates did not significantly vary based on different dental specialties, genders, or procedure durations. The dental students were vigilant about anticipating perforations at the end of procedures. More attention should be given to the right thumb and right index finger, as they are the most common sites of perforation. To mitigate cross-infection resulting from glove integrity loss during dental procedures, stricter glove requirements should be implemented, such as double gloving, frequent glove changes for vulnerable patients, and covering any wounds with plaster before donning gloves.

## Data Availability

The raw data supporting the conclusions of this article will be made available by the authors, without undue reservation.
